# A novel nonsense mutation in NPR2 gene causing Acromesomelic dysplasia, type Maroteaux in a consanguineous family in Southern Punjab (Pakistan)

**DOI:** 10.1007/s13258-020-00955-3

**Published:** 2020-06-06

**Authors:** Saima Mustafa, Zafrin Akhtar, Muhammad Latif, Mubashir Hassan, Muhammad Faisal, Furhan Iqbal

**Affiliations:** 1 Zoology Division, Institute of Pure and Applied Biology, Bahauddin Zakariya University, Multan, 60800 Pakistan; 2 Department of Zoology, Division of Science and Technology, University of Education Lahore, Multan Campus, Multan, Pakistan; 3 Institute of Molecular Biology and Biotechnology (IMBB), The University of Lahore, Lahore, Pakistan; 4 Faculty of Health Studies, University of Bradford, Bradford, UK

**Keywords:** AMDM, WES, Sanger sequencing, Western blot, 3D protein structure

## Abstract

**Background:**

Acromesomelic dysplasia, type Maroteaux (AMDM) is a rare skeletal dysplasia following autosomal recessive mode of inheritance and characterized by abnormal growth plates, short and abnormal bones in the extremities and spine.

**Objective:**

Present study was designed to report the molecular basis of AMDM in enrolled consanguineous family from Pakistan.

**Methods:**

A consanguineous family from Vehari District in Pakistan having multiple siblings suffering from AMDM was enrolled in present study. Whole exome sequencing (WES) approach was adopted to identify causative agent of AMDM. Human full length NPR2 gene and sequence with nonsense mutation was amplified by using Myc-tagged pXN vector and transformed in *E. coli* DH5α cells to confirm mutation. SDS-PAGE and Western blotting were done to confirm the production of truncated protein. Computational three dimensional structure generation through homology modeling approach was done to compare protein structure between patients and controls.

**Results:**

WES reveled a nonsense mutation (c.613 C>T, p.R205X) in exon 1 of NPR2 gene leading to premature termination codon in mRNA of NPR2 gene resulting in a truncated protein with 204 amino acid residues that was confirmed by SDS-PAGE and Western blotting. Sanger sequencing confirmed that mutation in all subjects and mutation followed Mendalian pattern of inheritance. Multiple sequence alignment by ClustalW revealed that mutated domain of NPR2 is conserved region. Proetin structure comparison revealed a significant structural part of NPR2 was missing in truncated protein as compared to control.

**Conclusion:**

We are reporting that a novel nonsense mutation (c.613 C>T, p.R205X) in exon 1 of NPR2 gene is causing AMDM in a consanguineous Pakistani family.

## Introduction

Acromesomelic dysplasias is a pathological condition during development during which skeletal elements under goes disproportionate shortening and usually middle parts of the forearms and legs are more affected (Bartels et al. 2004; Irfanullah et al. 2015). Acromesomelic dysplasia has three reported sub types based on phenotypic and radiological variations recorded in the patients: type Grebe (AMDG) (MIM #200700) (Umair et al. 2016), type Hunter and Thompson (AMDH) (MIM #201250) (Ullah et al. 2018) and Maroteaux type (AMDM) (MIM #602875) (Bartels et al. 2004).

Acromesomelic dysplasia, type Maroteaux (AMDM) is a rare skeletal abnormality with a frequency of about 1 in 1,000,000 birth and follows autosomal recessive mode of inheritance (Khan et al. 2012). In patients with AMDM can be identified by one year of age as they exhibit significant reduction in skeletal growth in the extremities and spines and having abnormal growth plates (Bartels et al. 2004). Acromesomelic dysplasia was mapped on chromosome 9p13-q12 (Kant et al. 1998) and Bartels et al. (2004) identified mutations in *NPR2*, encoding natriuretic peptide receptor B (NPR-B). NPR-B is a receptor for C-type natriuretic peptide (CNP) which is a paracrine and/or autocrine regulator of endochondral bone growth and affects intracellular secondary messenger, cyclic GMP (cGMP) production and action (Schulz 2005).

In present study we used a whole exome sequencing approach to identify the causative agent of AMDM in a Pakistani consanguineous family from Southern Punjab. Here we report that AMDM in this family was due to a novel missense point mutation in NPR2 gene indicating that a principal function of NPR2 is the regulation of skeletal growth during and after human development.

## Material and method

### Editorial policies and ethical considerations

All the experimental protocols and subject handling procedures were approved by the ethical committee of Institute of Pure and Applied Biology, Bahauddin Zakariya University Multan, Pakistan. Written informed consent was obtained from all subjects and/or guardians to use their data and pictures in publications (consent forms are enclosed as supplementary material).

### Blood and data collection

**Fig. 1:**
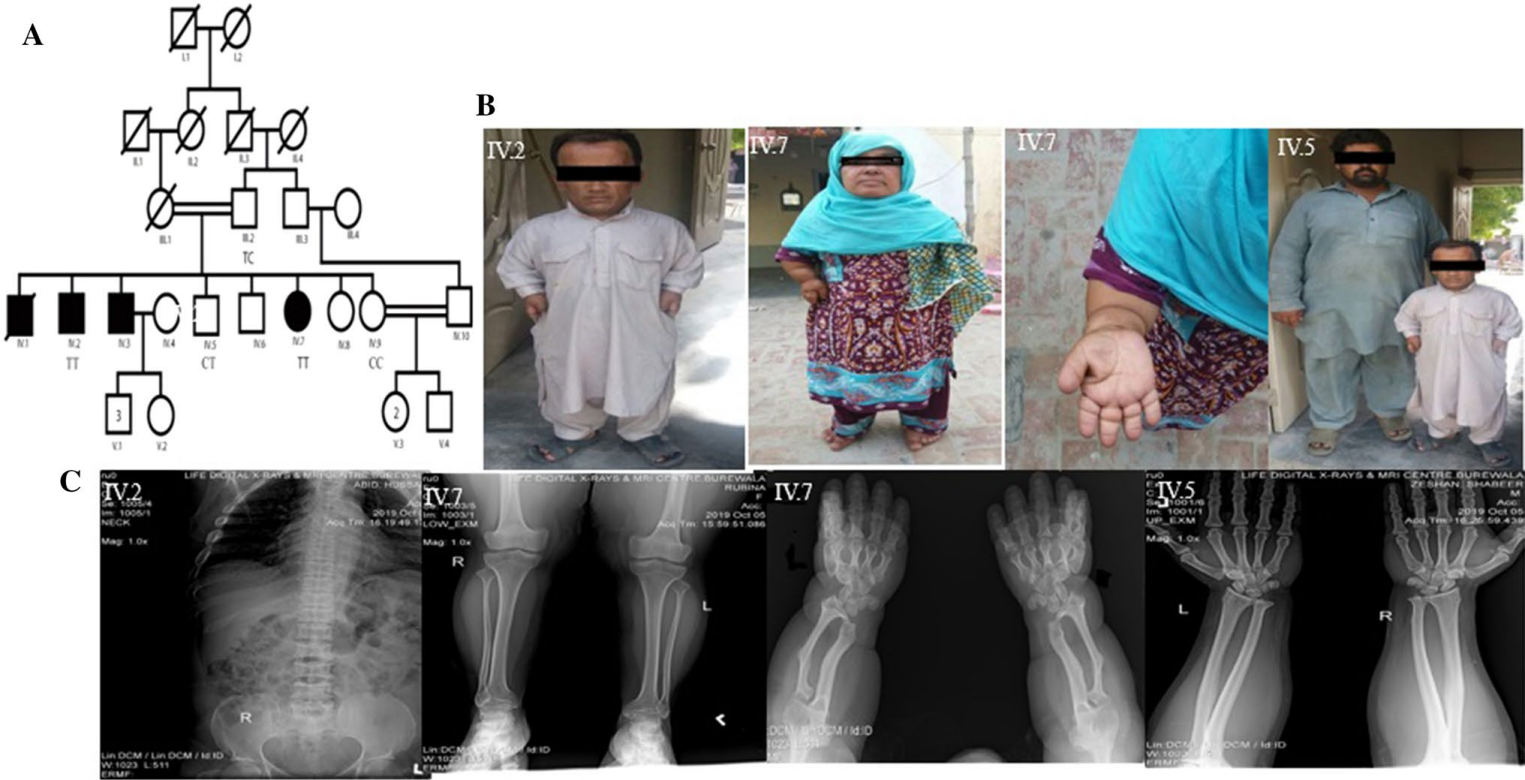
Pedigree and clinical manifestations. **a** Pedigrees of a consanguineous Pakistani family segregating autosomal recessive form of AMDM. Double lines are indicative of consanguineous union. Clear symbols represent unaffected individuals while filled symbols represent affected individuals. The diagonal line through a symbol is indicative of a deceased family member. **b** Affected individual IV.2 and IV.7 showing disproportionate mesomelic shortening of the arms. Individual IV.7 showing extremely short fingers with redundant skins. Individual IV.2 with his unaffected brother. **c** Radiographic features of AMDM in two patients and one control. Radiograph o vertebral column of an affected member IV.2 showing mild platyspondyly. Radiograph of IV.7 showing epiphysis of the radius, shortening of ulna, short and stubby metacarpels. Radiograph of IV.5 showing hands and lower arms

A consanguineous family was enrolled from Vehari District in Punjab (Pakistan) having multiple siblings suffering from AMDM (Fig. 1a). The clinical diagnosis of AMDM was based on radiographic criteria. Five blood samples were collected from family including two patients and three controls (Fig. 1a). Blood was sampled from median cubital vein and preserved in 0.5 M EDTA containing blood collection tube. Body weight was determined by using weight machine and height was measured with the measuring tape. Radiography was done for both control and patients at commercial diagnostic lab for appendicular skeleton, thoracic and spinal regions. A questionnaire was filled for each subject on the sampling site in order to collect epidemiological data associated with AMDM, if any.

### DNA extraction

DNA extraction was carried out by using commercial kit (Qiagen, Germany) following the instructions of the manufacturer.

### Whole exome sequencing

Four individuals (III-2, IV-7, IV-2, IV-5) from the enrolled family were selected for whole exome sequencing (Fig. 1a). 1 µg DNA were used for whole exome sequencing. Whole exome sequencing and data analysis were performed as previously (Zhou et al. 2014). Variants were annotated by ANNOVAR. Candidate variants were filtered to select those that were nonsynonymous or in splice sites within six base pairs of an exon, had less than 1% mutant allele frequency in the gnomAD, Kaviar and in-house database, and were co-segregated with the phenotype.

### PCR

To confirm the sequence change identified by the exome sequencing, exon 1 of NPR2 and its flanking intronic sequences were amplified by PCR from genomic DNA by using GTGGCCCGCTTTGCCTCCCA as forward primer and GCTCCCGAAACTTAATATCC as reverse primer. Polymerase chain reaction was carried out in a total volume of 50 µl. PCR reaction mixture consisted 0.4 µM deoxynucleotide triphosphate (dNTPs), 0.3 µM ul of each primer, 2X of buffer, 5 µl of DNA template at a concentration of 40 ng/µl, 1 µl of KOD FX taq polymerase (Toyobo, Japan). Amplification of DNA was processed in a DNA thermo cycler (Applied Biosystems, USA). Thermal profile conditions were initial denaturation at 94 °C for 2 min followed by 35 cycles of denaturation at 98 °C for 10 s, annealing at 60 °C for 30 s and extension at 68 °C for 45 s. Final extension was carried out 4 °C for 1 min PCR products were keep at 4 °C till their electrophoresis on 3% Agarose gel.

### Sanger sequencing

Sanger sequencing was used to confirm variant identified by WES as previously described (Zhou et al. 2014).

### Cloning of NPR2 gene

Human (full length) NPR2 gene was amplified by using specific primers to remove stop codon. PCR amplification was done using forward primer (ATATGCGGCCGCATGGCGCTGCCATCACTTC, *NotI* restriction site underlined) and reverse primer (ATTCGCGATCGCCAGGAGTCCAGGAGGTCC, *SfaA1* restriction site underlined). The PCR amplified DNA fragment was purified using the PCR purification kit (Axygene, China) and then digested with *NotI* and *SfaAI*. Myc-tagged pXN vector was also digested with same enzymes. Digested PCR product and vector was ligated using DNA ligase (Thermofisher, USA) following manufacturer protocol to create wild type. Full-length NPR2. Ligated product was then transformed into competent *E. coli* DH5α cells (Invitrogen, Carlsbad, CA, USA). Colonies grown on ampicillin LB agar were picked and were sequenced to confirm ligation of full length NPR2 gene with expression vector. Nonsesne mutation in NPR2 were generated by PCR-based mutagenesis using a site-directed mutagenesis method (Heckman and Pease 2007) by using the wild-type NPR2 expression construct. Specific Primers were designed to insert mutation in Myc-tagged wt full length NPR2. After amplification PCR products were purified and digested with DpnI following the manufacturer’s protocol. Finally digested product was transformed into competent *E.coli* DH5α cells (Invitrogen, Carlsbad, CA, USA). Colonies grown on ampicillin LB agar were picked and were sequenced to confirm mutation.

### Cell culture and transfection

HEK293A cells were cultured in Dulbecco’s modified Eagle’s medium (DMEM) (Thermo fisher scientific, USA) supplemented with 10% fetal bovine serum at 37 °C with 5% CO_2_. HEK293 cells were plated at a density of 1 × 10^5^ cells/12-well plate and cultured for a day so as to reach confluence. Transfection was performed using the Polyethylenimine (PEI) (Thermofisher scientific, USA) according to the manufacturer’s instructions. The cells were used for the experiments 48 h after transfection. Recombinant proteins into the cell culture medium were analyzed by SDS-PAGE of cell extracts from transfected cells followed by immuno-blotting.

### SDS–polyacrylamide gel electrophoresis and immunoblotting

Cells were lysed by incubation in 1 ml RIPA lysis buffer (50 mM Tris–HCl pH 7.4, 150 mM NaCl, 1 mM EDTA, 1% Triton X-100, 1% sodiumdeoxycholate, 0.1% SDS and 1% protease inhibitor). On ice for 15 min. The cell monolayer was removed, centrifuged at 15,000 × g at 4 °C for 20 min and the supernatant was removed for analysis. The supernatant was subjected to assay for protein concentration by Bradford method. The 11 μl (30 μg protein) of the supernatant was mixed with 4 μl sample buffer containing 62.5 mM Tris–HCl, pH 6.8, 2% SDS, 10% glycerol, 2% mercaptoethanol and 0.01% bromophenol blue and denatured at 95 °C for 5 min. The proteins were separated by SDS–polyacrylamide gel electrophoresis and transferred to PVDF membrane. Membrane was incubated in 5% Bovine serum albumin (BSA) as blocking reagent for 30 min to stop nonspecific binding of primary antibody. The membrane was washed with TBST buffer and incubated with a mouse monoclonal antibody against Myc-tag (1:1000; Cell Signaling Technology) as primary antibody for overnight. After washing with TBST buffer, the membrane was incubated with Mouse monoclonal HRP (1:1000; Cell Signaling Technology) antibody as secondary antibody for 1 h followed by washing with TBST buffer. The membrane was further incubated with chemiluminescent substrate and exposed to high-performance chemiluminescence film. The films were scanned and measured by image analysis software (Alpha view, Protein simple USA).

### Multiple sequence alignment analysis

Sequence of NPR2 protein for different species were download from ensemble (https://asia.ensembl.org/index.html). Multiple sequence analysis was performed by ClustalW.

### Model building of target proteins

The three dimensional (3D) structures of NAPR2 were designed computationally by fetching amino acids sequences from Uniprot Knowledge Database (ID: P20594). Therefore, a homology modelling approach was employed to predict 3D structures of wild and mutant NAPR2 structures. The automated Swiss modelling approach (https://swissmodel.expasy.org/) was employed to predict NAPR2. Two templates (1X6V and 1JDN) having sequence identity (78.20 and 31.04%) was selected to build the models. Mutation in protein results in de-stability in protein structure which may results in abnormalities in human (Hassan et al. 2017).

## Results

### Phenotype and radiographic findings

Affected individuals in this family exhibited features of acromesomelic dysplasia, type Maroteaux (AMDM). Fingers of the affected members were extremely short with redundant skin. Limbs showed marked shortening in the middle and distal segments. A skeletal survey revealed disproportionate mesomelic shortening of the arms, phalanges and metacarpal bones (Fig. 1b). Radiographs of affected individuals (IV-7, IV-2, IV-5) showed bilateral triangular distal epiphysis of the radius and relative shortening of the ulna. Meta carpels were short and stubby bilaterally. Mild reduction in the heights of vertebral bodies was noted in the thoracic and lumbar spine (mild platyspondyly) (Fig. 1c). Enrolled subjects with AMDM did not exhibited any neurologic impairment or any other consistent abnormality of any organ system outside the skeleton.

### NPR2 gene mutation

**Fig. 2:**
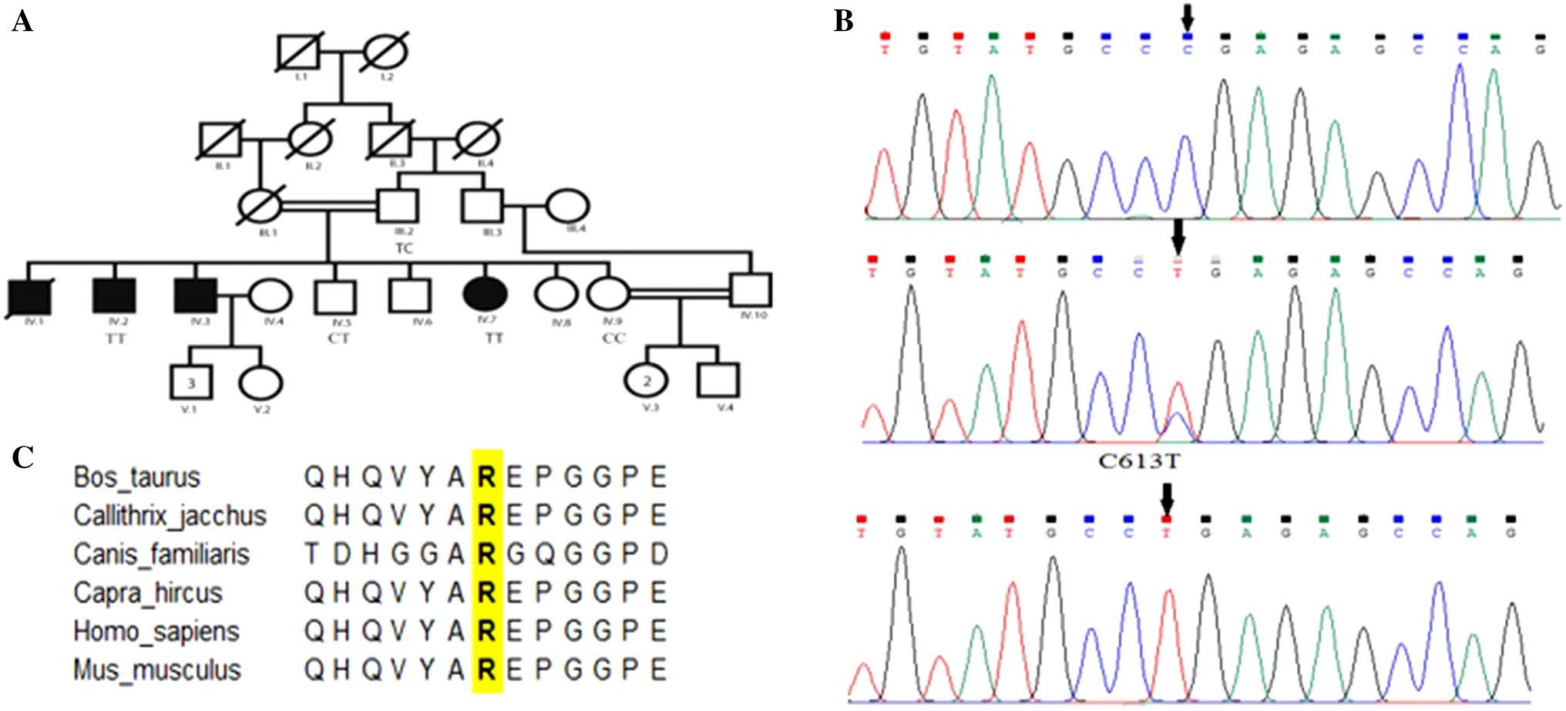
Chromatogram and ClustalW alignment of NPR2. **a** Four individuals (III-2, IV-7, IV-2, IV-5) were selected for whole exome sequencing. According to the mode of inheritance, individuals with normal height (III-2 and IV.5) carried heterozygous alleles (C/T) While affected individuals (IV.7, IV.2) carried homozygous mutant alleles (T/T). Mutation in six family members was confirmed by Sanger sequencing. **b** Chromatogram for NPR2 selected region showing c.613 C>T transition. **c** Multiple sequence alignment of NPR2 from six different organisms performed with Clustal showing p.R205X (shown in bold) conservation in diverse vertebral species

Whole-exome sequencing was performed in three Patients (IV-7, IV-2, IV-5) and their unaffected father (III-2). After filtering for novel and rare variants (allele frequency, < 1%), we identified approximately 4880 candidate variants in each trio. We hypothesized that the disorder could be caused by either de novo or recessive mutations. A single common candidate gene was identified only under the recessive model. All suspected variants were confirmed by Sanger sequencing. Sequence analysis of the gene *NPR2* detected a novel homozygous C to T transition at nucleotide position 613 (c.613 C>T, p.R205X) in exon 1 in affected individuals of enrolled family. While the normal siblings were either heterozygous or they had normal ‘C’ nucleotide for above mentioned mutation at position 613 in exon 1 (Fig. 2a, b). This mutation caused a premature termination of codon in mRNA sequence resulting in a truncated protein with 204 amino acid residues as compared to full length protein which is 1047 amino acids long. The variant was not observed in 100 normal control chromosomes or public databases.

ClustalW analysis revealed that Arginine and other amino acids of this domain are highly conserved in vertebrates (Fig. 2c) and any mutation in this region may have profound effects on the phenotype of the individuals.

### Full length and mutated NPR2 gene expression

**Fig. 3:**
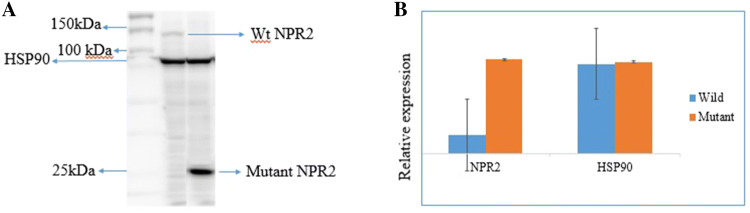
Western blotting and relative Protein expression. **a** 293T cells were transiently transfected with expression plasmids for the NPR2 target protein with a Myc tag. Cells were collected 48 h later, and equal amounts of the whole-cell lysates were subjected to immunoprecipitation with antibodies against the target protein. Lane 2 for wild type NPR2 protein, lane 3 for mutant target protein. Lane 1 protein molecular marker of 250 Kd. **b** Relative expression of proteins. Truncated NPR2 protein showing higher expression at 25kD as compared to full length wild NPR2. House keeping gene HSP90 showing similar expression in both plasmids (Wild type and mutant)

Whole-cell extracts from HEK 293 cells transfected with Myc-tagged PxN-NPR2 Wild and mutant constructs were resolved by SDS-PAGE and Western blotting. Western blot analysis confirmed that the mutant protein had significantly reduced mass (25kD) as compared to normal NPR2 protein with approximately 120 kDa mass (Fig. 3a, b).

### NAPR2 protein structure analysis

**Fig. 4:**
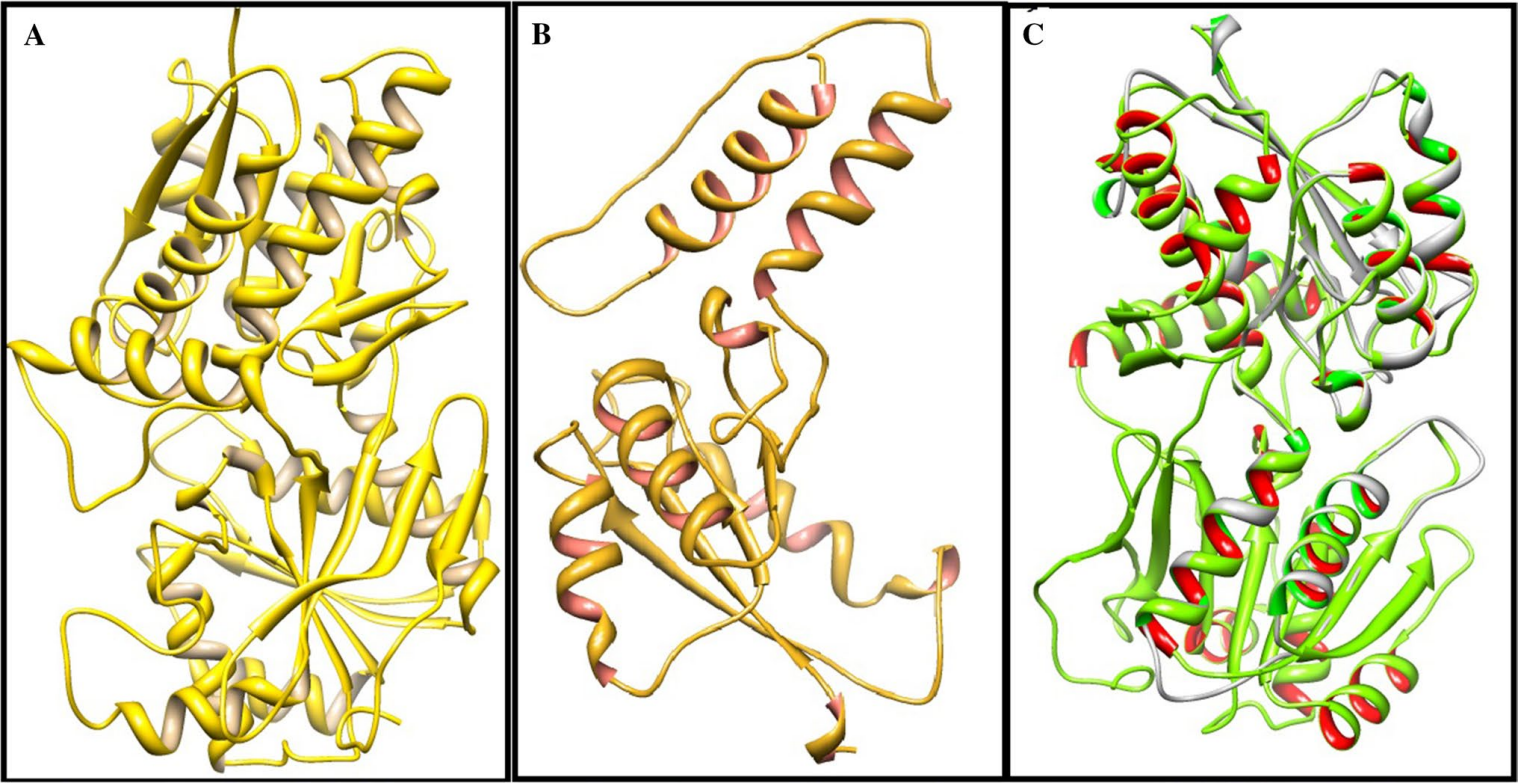
Predicted protein structures for NPR2.** a** Predicted protein structure for wild NPR2. **b** Predicted protein structure for mutant NPR2. **c** Superimposition of wild (green) and mutant (grey) structures (NAPR2) (color figure online)

In NAPR2 protein the sequence of wild and mutant protein showed marked difference in protein structure (Fig. 4). It has been observed from the superimposed model results, both predicted models align in perfect order and matched with respect to their structural parts as long as the two proteins are present but a significant structural part is missing in truncated protein as compared to control one (Fig. 4).

## Discussion

A consanguineous Pakistani family exhibiting short height and skeletal abnormalities was investigated in the present study (Fig. 1a). Diagnosis of AMDM in this family was based upon characteristic physical and x-ray findings in the affected individuals that resembled to those reported earlier in families of different ethnic origin with AMDM (Olney et al. 2006; Hachiya et al. 2007). By using whole exome sequencing approach, we identified a nonsense mutation (c.613 C>T) in exon 1of NPR2 causing AMDM in enrolled family (Fig. 1). This mutation caused a premature termination of codon in mRNA sequence resulting in a truncated protein with 204 amino acid residues.

Natriuretic peptide receptor (NPR) family consist of three members: NPR-A, NPR-B and NPR-C, that bind with natriuretic peptide hormones and regulate a number of physiological processes including cardiac growth, blood pressure (Kishimoto et al. 2001) and endochondral ossification (Tamura et al. 2004). NRP receptors act via production of the intracellular secondary messenger, cyclic GMP (cGMP) (Schulz 2005) that activates cGMP-dependent protein kinase II and inhibits MAPK pathway to promote the accumulation of extracellular matrix in the growth plate (Yasoda et al. 2004; Chusho et al. 2001; Teixeira et al. 2008). Both CNP and NPR2 are expressed in proliferative and pre-hypertrophic chondrocyte layers of the growth plate (Yasoda et al. 2007).

Some studies has already been documented reporting mutations in NPR2 gene in families with AMDM from different ethic background (Bartels et al. 2004; Olney et al. 2006; Hachiya et al. 2007). Bartels et al. (2004) had sequenced DNA from 21 families from various ethanic and geographical backgrounds (including four Pakistani families as well) affected by AMDM and found four nonsense mutations, four frameshift mutations, two splice-site mutations, and 11 missense mutations. Patients with AMDM had short stature, the shortening of the extremities, and the bowing of the fore arm. We observed a similar phenotype in ou enrolled subjects (Fig. 1). Bartels et al. (2004) used molecular modeling to examine the putative protein change brought about by each missense mutation. Three missense mutations were tested in a functional assay and were found to have markedly deficient guanylyl cyclase activity (Bartels et al. 2004).

Olney et al. (2006) had enrolled unrelated idiopathic patients with short stature from USA and identified seven heterozygous *NPR2* missense or splice site mutations all in the short stature patients, including one de novo splice site variant. They had reported that *NPR2* functional haploinsufficiency contributes to short stature and estimated a prevalence of *NPR2* haploinsufficiency of between 0 and 1/26 in people with idiopathic short stature.

Hachiya et al. (2007) enrolled a 28-year-old Japanese male presented with marked short stature and marked shortening in the middle and distal limb segments (similar phenotype is presented by subjects enrolled during present study). Direct sequencing of coding region of the *NPR2* gene of the family identified a novel missense mutation L658F in intracellular kinase homology domain (KHD) of NPR-B in homozygous and heterozygous states in the patient and his parents, respectively. The mutation conferred normal binding affinity for C-type natriuretic peptide but no discernible ligand-induced cGMP production. This study provided the first evidence that intact KHD of NPR-B is essential for skeletal development.

A couple of reports have already been documented on AMDM from Pakistan. Khan et al. (2012) had done sequence analysis of NPR2 in six Pakistani families suffering from AMDM. All the patients had disproportionate mesomelic shortening of the arms, phalanges and metacarpal bones and fingers were extremely short with redundant skin. Limbs showed marked shortening in the middle and distal segments (phenotype similar to those subjects reported in present investigation). They identified a novel missense mutation (p.T907M) in five families and a splice donor site mutation c.2986 + 2 T>G in the other family. Similarly in another study from Pakistan, Irfanullah et al. (2015) had investigated three consanguineous families segregating AMDM in an autosomal recessive manner. All the affected individuals were showing disproportionate short stature with shortening of middle and distal segments of the limbs. They reported two novel missense variants (p.Arg601Ser; p.Arg749Trp) in two families and a previously reported splice site variant (c.2986 + 2 T > G) in the third family.

Role of CNP has been well explored in rodent models. It has been reported that CNP knockout mice were of short stature than wild type while mice over expressing CNP had longer bones than control (Chusho et al. 2001; Yasoda et al. 2004). It has also been documented that exogenous administration of CNP to tibia cell culture of mouse and rat indicated that CNP can stimulate chondrocyte size and their proliferation (Yasoda et al. 1998; Mericq et al. 2000). Since CNP is able to increase chondrocyte proliferation, matrix synthesis, and cell hypertrophy in the growth plate, it is likely that each of these effects is mediated by signaling via NPR-B (Yasoda et al. 2004).

## Conclusion

In conclusion, we have reported a novel nonsense mutation (c.613 C>T, p.R205X) in exon 1 of NPR2 gene in a consanguineous Pakistani family. Review of literature has revealed a series of mutation in NPR2 gene from Pakistani families suffering from AMDM confirming that mutations in this gene can lead to AMDM in local population.

## Data Availability

All the data related with this project is available with the corresponding author and will be provided upon request.
